# The 2021 Ebola virus outbreak in Guinea: Mistrust and the shortcomings of outbreak surveillance

**DOI:** 10.1371/journal.pntd.0009487

**Published:** 2021-06-24

**Authors:** Manuel Raab, Emmanuelle Roth, Vinh-Kim Nguyen, Guenter Froeschl

**Affiliations:** 1 Division of Infectious Diseases and Tropical Medicine, University Hospital (LMU), Munich, Germany; 2 Department of Social Anthropology, Cambridge University, Cambridge, United Kingdom; 3 Department of Anthropology and Sociology, The Graduate Institute (IHEID), Geneva, Switzerland; Hospital Infantil de Mexico Federico Gomez, MEXICO

## Introduction

In February 2021, an outbreak of Ebola virus disease (EVD) was declared in the N’Zérékoré prefecture, located in the southeastern area of Guinea known as “Forest Guinea” [1]. This region is where the 2013 to 2016 West African epidemic—the largest Ebola epidemic to date—started. In the aftermath, Forest Guinea gained the status of a high-risk region for EVD and other viral haemorrhagic fevers [2]. Consequently, outbreak surveillance and preparedness planning have focused on preparing for a recurrence, such as the 2021 resurgence of Ebola.

Surveillance efforts aim for rapid containment through early detection, specific treatments, and an efficient vaccine. In fact, efforts deployed in the region appear to have detected the current resurgence within a time frame of somewhat less than 1 month. Local communities view the current response through the lens of the earlier outbreak and the preparedness efforts that followed. The response to the 2013 to 2016 epidemic in Forest Guinea was characterised by deep mistrust and violence. In its aftermath, preparedness efforts have been met with scepticism in the population, including healthcare workers, with regard to the ability of the public health system being able to manage future outbreaks effectively.

## Epidemic experiences and outbreak preparedness in Forest Guinea

The authors MR and ER researched post-Ebola outbreak preparedness in Forest Guinea while living there from 2017 to 2019.

Forest Guineans told them of their worries that a new outbreak may again take a heavy toll. The case fatality rate of the 2013 to 2016 EVD outbreak was reportedly 67% for Guinea [3]. Currently, the population is even more concerned with the impact on their livelihoods, already strained by the economic effect of the Coronavirus Disease 2019 (COVID-19) pandemic. Furthermore, Forest Guineans remember how during the last Ebola epidemic, their “culture”—food habits and burial customs—was blamed as a potential driver of the outbreak, and heavy-handed measures repressed dissenting views. The ill were isolated inside Ebola treatment centres that resembled internment camps for political opponents. Many were buried in the absence of relatives, anonymously, and in plastic bags [4]. Outbursts of violence made victims among both the response personnel and involved populations. In October 2014, senior officials were murdered during a sensitisation event in Womey, a village north of N’Zérékoré, after which the military retaliated against the inhabitants [5].

Such episodes of “resistance” should be understood against a background of historical and political inequality [6]. Resistance was the exception rather than the rule, and widespread cooperation by a majority did help to bring the outbreak in Forest Guinea under control. But these epidemic experiences have come to structure local expectations of Ebola outbreaks in general: People perceive that the health system may not save their lives nor recognise their ways of caring for their kin, that authorities tend to blame local cultural preferences, and that rumours and political tensions may escalate into deadly conflicts.

The earlier lessons of the 2013 to 2016 epidemic demonstrated how weaknesses in primary healthcare could foster the uncontrolled spread of infectious diseases [7]. Subsequently, Guinea’s healthcare system received development support, explicitly aimed at “restoring” trust in their health facilities. Medical devices and materials were donated, health administrations were equipped, and policies for the retention of the health workforce in the countryside were implemented. International organisations and national institutions also invested in mechanisms for disease surveillance and outbreak response: The post-Ebola resilience plan for Guinea allocated 18% of the national healthcare budget to that sector [8].

The National Agency of Health Security has been implementing the novel disease surveillance mechanisms in order to “guarantee a healthy environment without epidemics” [9]. In the N’Zérékoré region, health risks were mapped. Ebola survivors were included in observational cohort studies, which monitored their health and signs of viral activity [10]. Regional and prefectural outbreak response teams were trained to conduct epidemiological investigations. More than 30 specialised centres for the treatment of diseases with epidemic potential (CTEPI) were erected to isolate and treat patients with infectious diseases. Regional laboratories were equipped with PCR machines for the detection of viral haemorrhagic fevers. Since 2014, research on the ecological reservoir of Ebola has mostly targeted Forest Guinea, with many teams coming to sample wildlife [11]. Forest Guinea became a testing ground for experimenting preparedness mechanisms and researching the ecological and immunological traces left by the disease.

## The social legacy of outbreak preparedness

Between February 8 and 13, 2021, 5 patients from a single family were treated for febrile illness in N’Zérékoré regional hospital. All patients had attended the burial of the presumed index case on February 1 in Gouéké, a town 50 kilometres from N’Zérékoré. The 5 patients showed unspecific symptoms upon admission: fever, diarrhoea, and bleeding. As per national case definition criteria, these symptoms are suspicious for viral haemorrhagic fever. On February 12 and 13, 2 of them died without having been tested for EVD. Their deaths raised concerns, and the 2 remaining patients were tested. The third patient had already self-discharged on February 11 and travelled to the country’s capital. He was located and tested in the capital on February 13. These 3 patients became the first laboratory-confirmed EVD cases in Guinea since the 2013 to 2016 epidemic [12]. In the subsequent 2 weeks, 3 healthcare workers from the N’Zérékoré regional hospital and 2 healthcare workers from other facilities also tested positive for EVD [13]. At the time of writing (May 12, 2021), 23 EVD cases (16 confirmed and 7 probable) have been reported in Guinea [14].

Healthcare workers are generally aware of EVD screening guidelines, and the N’Zérékoré regional hospital boasts one of the 2 viral haemorrhagic fever laboratories in Forest Guinea [15]. But staff rarely test patients for EVD [16]. Figures for the entire country underscore the low uptake of viral haemorrhagic fever testing since the outbreak: In 2018, the Guinean outbreak surveillance and response programme reported and tested only 16 suspect cases for EVD [17]. In contrast, wildlife sampling for viral surveillance has sharply increased in Guinea since 2016. Next to French, German, and Russian wildlife sampling initiatives, the USAID-funded project PREDICT alone sampled more than 4,700 animals between 2016 and 2020, but most of these samples were tested in overseas laboratories [11].

In the course of his research at public healthcare facilities, patients told MR that they fear the consequences of an EVD suspect case diagnosis, and healthcare workers worried about being blamed if they declare suspect cases. For instance, we witnessed a patient leaving the N’Zérékoré hospital’s inpatient ward after being identified as an EVD suspect case. He died and was buried in his village the next day. Since he had not been tested, the hospital staff did not judge appropriate to report him as a suspect case and be blamed for the patient’s “escape.” In another instance, the family of a potential suspect case—who included military personnel—told hospital staff not to test a patient. As word spread that the patient’s symptoms resembled those of EVD, nurses refused to provide further treatment to the patient. Such events are not reported to health authorities. It is thus both impressive and surprising that the early case cluster in February was detected in such a short time by the Guinean surveillance system, considering that viral haemorrhagic fever screening in patients is often problematic in the local clinical setting.

**Fig 1:**
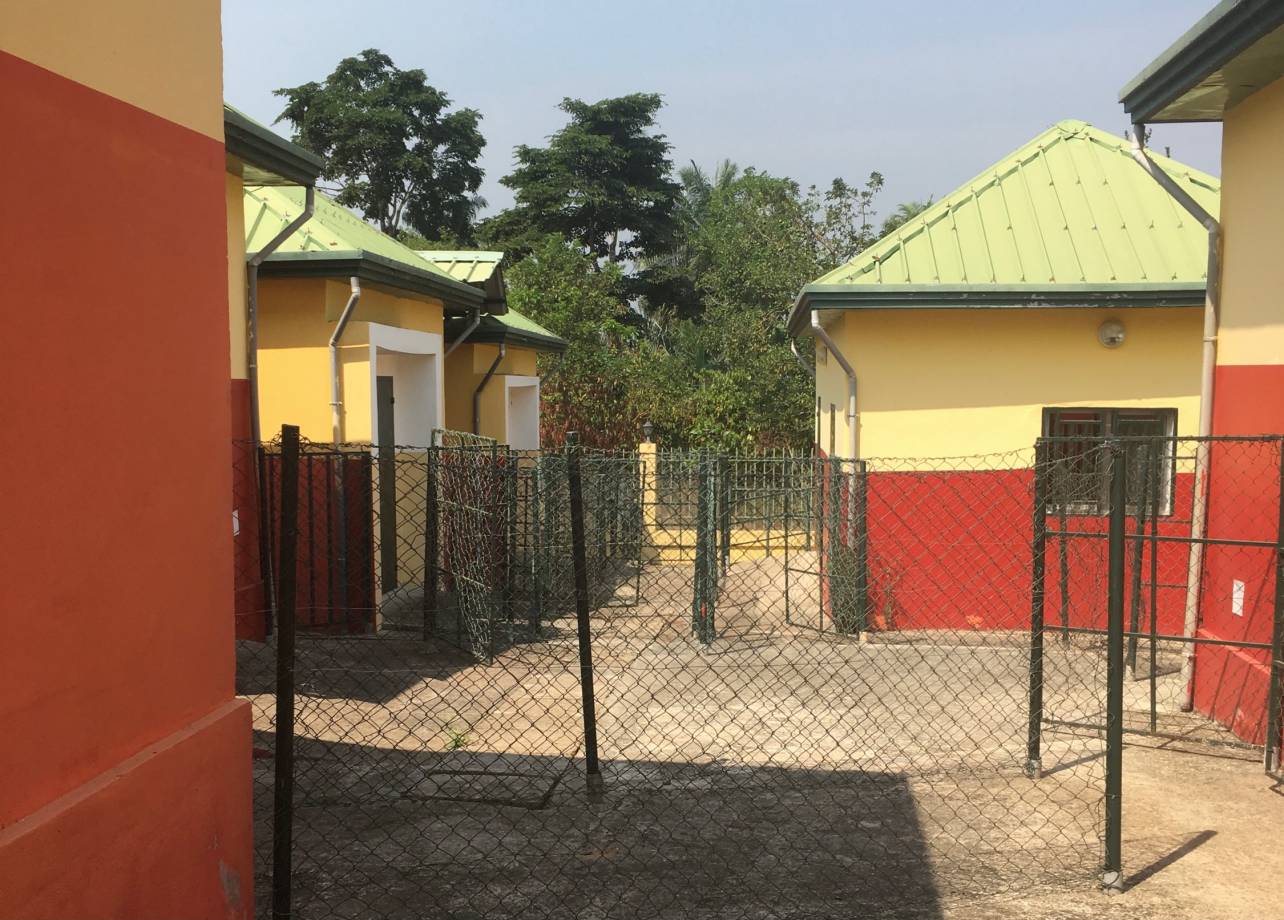
N’Zérékoré CTEPI, 2019. Photo taken by the author MR.

MR and ER observed that suspicions extend beyond the hospital surveillance system. The entire post-Ebola outbreak apparatus arouses reactions of fear and avoidance in the population, including healthcare workers. Since 2017, very few infectious disease patients have been isolated in CTEPIs, so that by mid-2019, the health administration attempted to repurpose them for primary healthcare. While we believe that these are important steps towards community acceptance of CTEPIs, locals in Forest Guinea have been reluctant to visit these clinics, whose architecture is alike that of Ebola treatment centres (Fig 1). Furthermore, programmes for the follow-up of Ebola survivors detected Ebola viral material in semen, but for fear of stigmatisation, these results were disregarded. Veterinary surveillance workers seeking samples from wildlife were initially chased away, as rumours spread that they were injecting pathogens into the local fauna. Anticipating hostility towards their activity, some samplers relinquished full body protection or tried to dissimulate their sampling activity.

Outbreak preparedness and surveillance measures have contributed to the early identification of the current outbreak. But mistrust lingers, accepted, expected, and corroborated by the everyday practice of surveillance and outbreak preparedness measures. It is an important legacy of a historically unprecedented epidemic that crystallised a complex sociopolitical crisis in Forest Guinea.

## Conclusions

Contact tracing, safe burials, and a ring vaccination campaign are under way in Forest Guinea to contain the current Ebola outbreak. There is a risk that the effectiveness of these measures during current and future outbreaks will be mitigated if healthcare workers and the local population mistrust the outbreak response. Indeed, mistrust in health actors and structures has not been fully addressed; its consideration has rather been integrated into the practice of disease surveillance.

Some aspects of this post-Ebola legacy of mistrust in Forest Guinea can be addressed. For example, care should be the primary goal of CTEPIs and Ebola treatment centres, rather than isolation. Furthermore, in the light of new evidence suggesting that the outbreak was sparked by a latent infection in an EVD survivor from the 2013 to 2016 epidemic and not a zoonotic spillover event [18], research, risk communication, and outbreak surveillance should pay more attention to humans. Screening for EVD in patients with conspicuous symptoms should become a clinical routine practice, accepted by patients as well as healthcare staff. Its acceptance requires respectful and honest communication aimed at establishing trust, rather than avoiding fearful and suspicious reactions. Finally, responders and healthcare personnel should recognise their role in the coproduction of mistrust and not only attribute mistrust to “resistant” communities or individual patients.
